# Metagenomic analysis of the soil microbial composition and salt tolerance mechanism in Yuncheng Salt Lake, Shanxi Province

**DOI:** 10.3389/fmicb.2022.1004556

**Published:** 2022-09-26

**Authors:** Feifeng Zeng, Yonghong Zhu, Dongling Zhang, Zengqiang Zhao, Quansheng Li, Panpan Ma, Guoli Zhang, Yuan Wang, Shenjie Wu, Sandui Guo, Guoqing Sun

**Affiliations:** ^1^Biotechnology Research Institute, Chinese Academy of Agricultural Sciences, Beijing, China; ^2^Cotton Research Institute, Shanxi Agricultural University, Shanxi, China; ^3^Xinjiang Academy of Agricultural and Reclamation, Xinjiang, China

**Keywords:** Salt Lake, soil microorganism, metagenome, salt tolerance mechanism, microbial composition

## Abstract

The soil in Yuncheng Salt Lake has serious salinization and the biogeographic environment affects the composition and distribution of special halophilic and salt-tolerant microbial communities in this area. Therefore, this study collected soils at distances of 15, 30, and 45 m from the Salt Lake and used non-saline soil (60 m) as a control to explore the microbial composition and salt tolerance mechanisms using metagenomics technology. The results showed that the dominant species and abundance of salt-tolerant microorganisms changed gradually with distance from Salt Lake. The salt-tolerant microorganisms can increase the expression of the Na^+^/H^+^ antiporter by upregulating the Na^+^/H^+^ antiporter subunit mnhA-G to respond to salt stress, simultaneously upregulating the genes in the betaine/proline transport system to promote the conversion of choline into betaine, while also upregulating the trehalose/maltose transport system encode genes to promote the synthesis of trehalose to resist a high salt environment.

## Introduction

Salt lake refers to a salty water body with salt content > 50 g/L, which occurs under natural processes through the continuous addition of salts and other complex substances and the action of geological conditions (Lindsay et al., 2019). Such water bodies contain important chemical components, making them an important source of various inorganic salts for industry (Xu et al., 2020; Zhou et al., 2020; Sun Y. et al., 2021). Yuncheng Salt Lake in Shanxi Province, also known as the dead sea of China, is in the southern suburb of Yuncheng city, Shanxi Province in eastern China (110°07′∼110°50′E, 34°04′∼34°54′N) (Zhang et al., 2020). It is the third largest, natural, inland sodium-sulfate lake in the world. Yuncheng Salt Lake is rich in mirabilite, salt, and other substances, so nearby soil salinization is serious, surrounding vegetation is sparse, and the plants near the lake are mostly perennial herbs such as algae and reeds (Wang et al., 2014; Gao et al., 2021). The concentration of Mg^2+^, Cl^–^, Na^+^, and SO_4_^2–^ in surface brine is higher than that of other ions, so it is considered a typical quaternary aqueous salt system of Mg^2+^, Cl^–^, Na^+^, SO_4_^2–^, and H_2_O (Huan et al., 2019). It contains important mineral and biological resources. Research related to the lake has primarily focused on the human environment, salt tolerant plants, and salt production, yet there are few reports on the microbial diversity and biological mechanisms of salt tolerance in this area (Huan et al., 2019; Ming et al., 2020; Gao et al., 2021). At the same time, biogeochemical properties affect the composition and distribution of special halophilic and salt-tolerant microbial communities in this area (Kanik et al., 2020). In view of the special nature of the saline environment of Yuncheng Salt Lake, the high-salt soil and the salt lake itself provide excellent opportunities for research on the isolation and screening of salt-tolerant microorganisms and exploring mechanisms of biological salt tolerance.

Halophilic microorganisms are widely distributed in high-salt environments such as oceans, salt lakes, salt fields, and pickled foods (Banciu et al., 2019; Montalvo-Rodríguez and Maupin-Furlow, 2020). As a microbial resource with diverse application prospects, halophils have attracted extensive attention at home and abroad because of their special physiological characteristics, metabolic capabilities, interactive community composition, and genetic resources (Dumorné et al., 2017; Hasanzadeh et al., 2020). Halophiles require a high salt concentration to stabilize their cell structure and maintain a proper cellular concentration of K^+^ ions. According to the dependence of Halophilic microorganisms are divided into five groups according to their dependence on salts, including non-halophilic, slightly halophilic, moderately halophilic, borderline extremely halophilic, extremely halophilic, and halotolerant microorganisms (Kushner, 1978). As a special new microbial resource, the study of halophilic organisms has important theoretical significance in the fields of biological evolution, biological adaptation to extreme environments, and practical opportunities and applications (Dubey and Mishra, 2021; Orhan, 2021). Therefore, special compounds found in halophiles were widely studied by microbiologists in the 1980s and have since been studied in the fields of biological theory and practical application of halophilic compounds (Cazzulo et al., 1973; Onishi and Hidaka, 1978; Masahiro, 1980). At present, halophilic compounds can be used in fermentation production processes. Because they offer the ability to resist the stress of a high salinity environment, these compounds can assist in high salinity fermentation processes and reduce production costs (Mokashe et al., 2018). At the same time, halophilic compounds also play an important role in the treatment of high-salt wastewater (Castillo-Carvajal et al., 2014). Using the purple membrane of extreme halophiles may also lead to the development of new technical products in the electronics field. Halophilic microorganisms also produce substances with special, bioactive properties (Díaz-Cárdenas et al., 2020; Martínez-Pérez et al., 2020). Therefore, research on halophilic compounds has certain theoretical and practical value. Current research on halophilic mechanisms is particularly important because microorganisms that live in extreme environments possess unique metabolic capabilities and functions (Chung et al., 2019). Microbial salt tolerance is of considerable interest because: (1) Specific types of eubacteria and haloarchaea maintain high cellular ion concentrations (K^+^, Na^+^) to sustain a constant osmotic pressure. Understanding the mechanisms that allow this process may offer interesting development opportunities; (2) Salt tolerant microorganisms stabilize osmotic pressure by maintaining a content of solutes with high biological compatibility in cells, which adjusts with variations in extracellular environment (Plemenitaš et al., 2014; Li et al., 2021). This, present research on microbial salt tolerance mechanisms is focused on osmotic pressure regulation. However, the specific pathways and gene regulation of salt tolerance mechanisms remain unclear.

In conclusion, Yuncheng Salt Lake, as a typical inland salt lake with considerable soil salinization, is an ideal resource for studying the diversity and functional expression of halophilic microorganisms, especially considering how little research has been conducted on its halophilic microbial community. The unique salt environment endows Yuncheng Salt Lake with a unique halophilic flora. Further study on the diversity of halophilic bacteria in Yuncheng Salt Lake is of great significance to the exploration of mechanisms of halophilic microbial physiology. The Lake represents a microbial resource with great basic research value and application prospects, considering that halophils have attracted extensive attention because of their unique physiological structure, metabolic capabilities, population diversity, genetic capacity, and functional metabolites. Therefore, this study focused on Yuncheng Salt Lake as an exceptional natural resource in southern Shanxi. This research focused on measuring the physical and chemical properties of the saline alkali soil of Yuncheng Salt Lake and the analyzing the diversity, composition, and salt tolerance mechanisms of salt-tolerant bacteria by using macrogenomic technology, this study lays a foundation for further understanding of the status of microbial resources in Salt Lake and the development of its salt-tolerant and halophilic microorganisms. This study provides a theoretical basis for the further development of industrial applications and mechanisms.

## Materials and methods

### Sample collection and specific soil sample information determination

**FIGURE 1:**
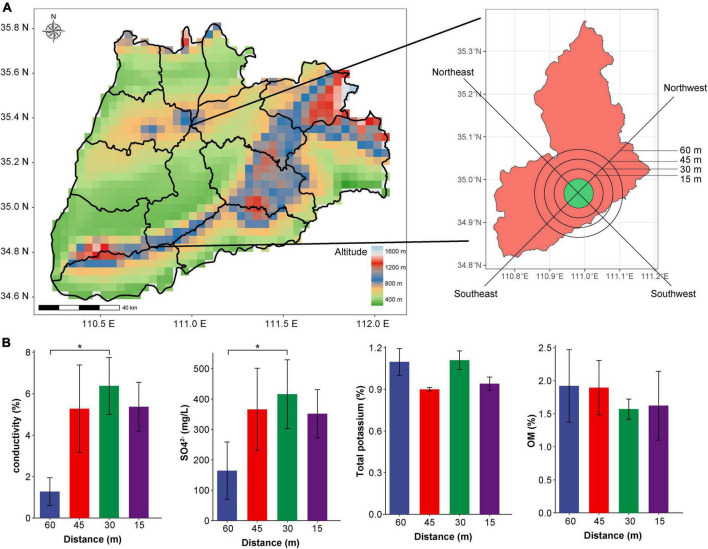
Satellite cloud image and physical and chemical indices of soil sampling points in Yuncheng Salt Lake. **(A)** Satellite cloud image of Yuncheng Salt Lake, Shanxi Province, China; **(B)** represents the changes in electrical conductivity, SO4^2–^, total potassium and OM content in soil at different distances (15, 30, 45, 60 m) from the lake shore. Data were analyzed by one-way ANOVA (*p < 0.05).

Soil samples were collected from the area surrounding Yuncheng Salt Lake in Shanxi Province. A satellite cloud picture of the Salt Lake soil is shown in Figure 1A. Soil was sampled for experimental analyses at intervals of 15 m, 30 m, 45 m, and a non-salt soil sample was collected at 60 m from the lake as a control. Samples were collected at the distances mentioned above proceeding to the southeast (SE), northeast (NE), southwest (SW) and northwest (NW) direction from the Salt Lake shore. After removing impurities from the soil surface, sample collection at a depth of about 8 cm, and take 500–800 g of soil for each sample. All 48 samples collected included three replicates at each location. The collected samples were stored in sterile plastic bags, transported to the laboratory on ice and frozen at −80^°^C y on ice and frometagenome sequencing analysis. At the same time, the physical and chemical properties (pH, OM, SO_4_^2–^, electrical conductivity, and total potassium) of the collected soil samples were determined refer to the method of Peng et al. (2021).

### Deoxyribonucleic acid extraction and sequencing

Soil genomic DNA was extracted from a small amount of soil by using the product manual provided by the Magnetic Soil and Stool DNA Kit (DP712, TIANGEN, China) (Zhu et al., 2021). The total DNA sample was sonicated to a fragment size of 350 bp and then used for library construction with the NEBNext^®^ Ultra™ DNA Library Prep Kit for Illumina (NEB, USA) following the manufacturer’s recommendations. The library preparations were sequenced on an Illumina NovaSeq 6000 platform and paired-end reads were obtained after pre-treatment.

### Bioinformatics analysis of the metagenome from soil samples

SOAPdenovo software was used for assembly analysis, and gene prediction and abundance analysis were performed based on the assembly results. Genes were compared with functional databases, and unigenes were compared with bacteria, fungi, archaea, and viruses extracted from the NR database (version: 2018.01) of NCBI using diamond software. Species annotation and abundance results were obtained, and the top 10 species with the largest relative abundance were selected. The R language tool was used to count the number of species, functions, or genes shared or unique in multiple samples. The “vegan” package of R (version 4.0.4) was used for PCoA (principal component analysis) statistical analysis. Alpha diversity analysis was conducted with QIIME1 software. The various species among different groups were identified using the rank sum test, and the species with significant differences between groups were screened by the “randomForest” and “pROC” packages of R. The weighted gene correlation network analysis (WGCNA) was conducted with R software and the co-expression network construction by the Gephi. The KEGG database was used to predict and analyze microbial gene expression functions. All statistical analyses and graphing were performed using R software.

## Results

### Determination of physical and chemical indices of soil sampled from Salt Lake

Through the measurement of pH, OM, SO_4_^2–^, electrical conductivity and total potassium in the soil near the salt pond, we found that there was no significant difference in the content of total potassium, OM, and pH with the change in distance (Figure 1B and Supplementary Figure 1A). Conductivity and concentration of SO_4_^2–^ at 60 m was significantly lower than that at 30 m (*P* < 0.01, Figure 1B), indicating that the soil conductivity in the salt lake area was significantly higher than that in the non-salt lake area, which was related to the high Na^+^ and Mg^2+^ ions in the salt lake area. In addition, Supplementary Figure 1B shows that the conductivity increases in direct relation to closeness to the lake shore; conductivity was lowest at 60 m and highest at 30 and 15 m. The various sampling directions have little impact on changes in conductivity, indicating that the soil conductivity changes significantly with the change in distance, reflecting the change in salt ion concentration in soil, which has a considerable impact on the composition of the soil microbial community. Therefore, we investigated the diversity of microorganisms in the soil according to distance from the lake shore (60, 45, 30, and 15 m).

### Analysis of the overall microbial composition in Salt Lake soil samples

**FIGURE 2:**
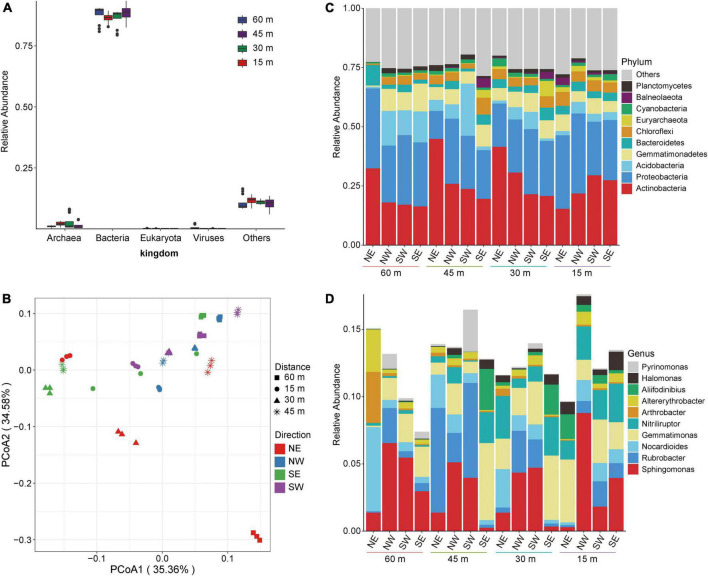
Microbial composition of soil in Yuncheng Salt Lake. **(A)** Relative abundance composition of soil microorganisms at the kingdom level. **(B)** PCoA (principal coordinate analysis) based on Bray–Curtis at the genus level. **(C)** The relative contribution of the top 10 phyla at different distances (15, 30, 45, 60 m). **(D)** The relative contribution of the top 10 genera at different distances (15, 30, 45, 60 m). *NE northeast, NW northwest, SE southeast, SW southwest.*

We extracted, sequenced, and analyzed the collected soil samples using metagenomic technology. A total of 313,696.71 Mbp of data was obtained by metagenomic sequencing, and a total of 10,942.01 Mbp of scaffolds were obtained after mixed assembly (Supplementary Table 1). The microbial composition of the soil samples was primarily bacteria (more than 75%) according to species annotation (Figure 2A). Therefore, we subsequently focused on a detailed analysis and discussion of the overall distribution and composition of the bacterial community. To explore the overall composition of microorganisms in different salt lake soils, we conducted principal coordinate analysis (PCoA) at the genus level based on the Bray–Curtis distance matrix. Figure 2B shows that the microbial composition of soil bacteria varied according to distance from the lake shore. Among the microbial community assessed, the PCoA1 distribution showed the largest contribution with recovered changes of 35.36%, while the PC2 distribution recovered changes of 34.58%. These results show that the dominant bacteria changed with distance from the lake shore indicating a direct relationship with the changes that we found in soil salt content which, therefore, directly influences the shape of the soil microbial community.

Thus, to understand the specific distribution and composition of microorganisms in Salt Lake soil samples, we further analyzed the composition of the flora at the phylum level and the genus level. Figure 2C shows that the soil samples in each group are composed of *Actinobacteria, Proteobacteria, Acidobacteria, Gemmatimonadetes, Bacteroidetes*, and *Chloroflexi.* In soils to the northeast of Salt Lake, the abundance of *Actinobacteria* and *Bacteroidetes* at 15 m were lower than those at 30, 45, and 60 m (Figure 2C), which increased the relative abundances of *Gemmatimonadetes* and *Chloroflexi*. At the same time, compared with 15 m and 60 m, the relative abundances of *Actinobacteria* at 30 and 45 m increased, and the relative abundances of *Proteobacteria* decreased accordingly. These results show that the soil microbial composition at 15 m from the lake shore is significantly different from that at 30 and 60 m away, and from non-salt lake soil. That is, the microbial composition and structure in the soil near the Salt Lake area changed under the influence of a high salt environment, which significantly promoted the proliferation and growth of salt-tolerant bacteria. In the areas to the northwest and northeast of the Salt Lake shore the soil microbial composition did not change significantly at the phylum level along the sampling distance gradient.

The analyses at the genus level showed that the soil samples of each group were composed of *Sphingomonas, Rubrobacter, Nocardioides, Gemmatimonas, Nitriliruptor, Arthrobacter, Alterythrobacter*, and *Aliifodinibius* (Figure 2D). There were different dominant groups of salt-tolerant bacteria proceeding in different directions from the lake shore. In the soil to the northeast of Salt Lake, the dominant bacteria at 15 m were *Gemmatimonas*, which was significantly higher than that in the soil samples collected in the other three directions. *Gemmatimonas* decreased at 30 m, and the relative abundance of *Nocardioides* and *Nitriliruptor* gradually increased to become dominant bacteria. *Rubrobacter* was the dominant bacterial genus at 45 m to the northeast of the lake shore, and the abundance of other flora decreased. The dominant bacteria in the soil to the northeast of the Salt Lake at distance of 60 m were *Nocardioides.* That is, from 60 to 15 m away from the lake shore the composition of the soil microflora gradually changed from *Nocardioides and Rubrobacter* to *Gemmatimonas* and *Nitriliruptor*. This may be related to the metabolism and regulatory mechanism of the flora adapting to external high osmotic pressure under high salt stress. The dominant bacteria to the northwest were *Sphingomonas* and *Rubrobacter*. At the same time, *Rubrobacter* showed a gradual downwards trend from 45 to 15 m from the lake shore, while *Sphingomonas* gradually increased and reached the maximum abundance level at 15 m to the northwest. The southwest variation trend was the opposite; *Sphingomonas* reached a minimum abundance level at15 m from the lake shore in the southwest, which was lower than that at 30 and 45 m. The soil microbial composition to the southeast of the lake shore, the dominant bacteria at 30 and 45 m were *Gemmatimonas, Nitriliruptor*, and *Aliifodinibius*. These results show that the dominant salt-tolerant bacteria varied in different directions from the Salt Lake shore.

### Significant differences in microorganisms in Salt Lake soil

**FIGURE 3:**
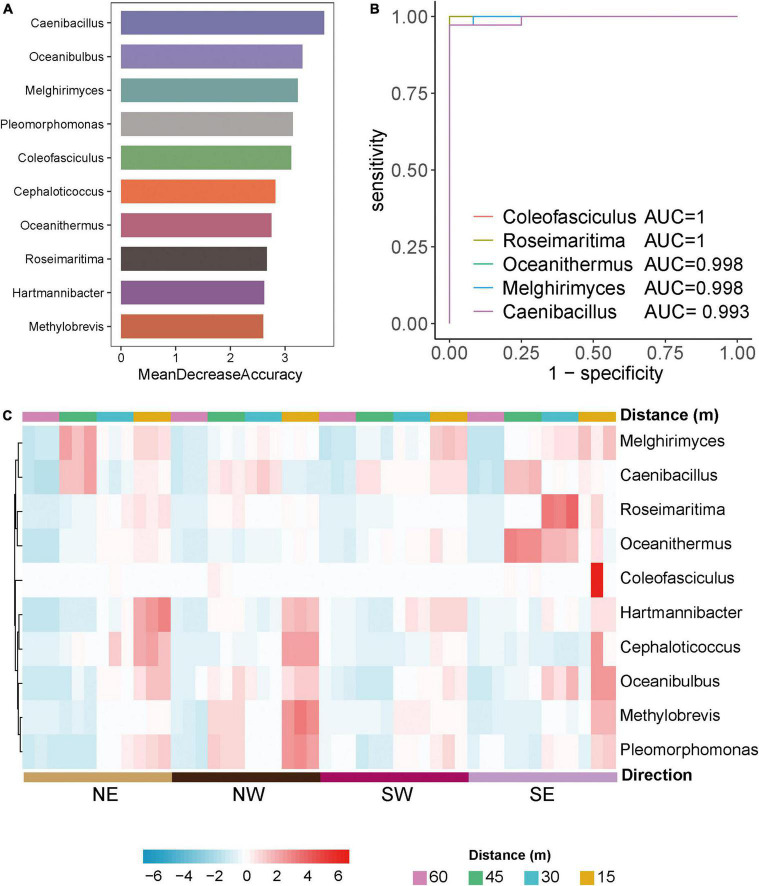
Differential microorganisms in Salt Lake soil. **(A)** The 10 most discriminant genera in the models classifying different distances of bacterial microorganisms at the genus level based on the random forest method. The bar lengths indicate the importance of the variable. **(B)** Evaluation of predictive grouping ability of soil microbiota markers based on receiver operating characteristic (ROC) curve analysis. Draw the curve with sensitivity as the ordinate. The closer the ROC curve is to the upper left corner, the higher the accuracy of the test. If the area under the ROC curve (AUC) value is 1.0, it reflects the perfect distinction between the two groups, and there is no prediction error. Higher accuracy when AUC is above 0.9. **(C)** Heatmaps of the 10 most discriminant genera at different distances (15, 30, 45, 60 m) and directions (NE, NW, SW, SE) in the model classification.

To explore differences in biomarkers between the microflora at various distances from the lake shore, we developed a random forest model based on the differential genera with a relative abundance > 0 in at least 95% of the samples of these groups (Edwards et al., 2018). The optimal model utilized 10 genera that provided the best discriminatory power (Figure 3A). These genera in the optimal model were members of *Caenibacillus, Oceanibulbus, Melghirimyces, Melghirimyces, etc.* We developed a receiver operating characteristic (ROC) curve and calculated the area under the ROC curve (area under the ROC curve, AUC) to evaluate the grouping ability of bacterial marker predictions and to explore which bacteria had the best diagnostic value (Figure 3B). The closer AUC was to 1, the better the prediction effect was. The results showed that the AUCs of *Coleofasciculus, Roseimaritima, Oceanithermus, Melghirimyces*, and *Caenibacillus* were 1, 1, 0.998, 0.998, and 0.993, respectively, indicating that these bacteria had excellent predictive power as biomarkers.

Based on these bacterial markers screened by the above random forest and ROC curve model, we created a heatmap of the relative abundance of bacteria at various distances and directions from the lake shore. Figure 3C shows that the model’s prediction results are accurate and that there are specific bacterial markers at various distances and in different directions from the lake shore for example, *Roseimaritima* and *Oceanithermus* had high abundance and enrichment to the southeast at 30 and 45 m, *Melghirimyces* and *Caenibacillus* had high abundance to the northeast at 45 m, and *Coleofasciculus* was differentially enriched at 15 m to the southeast.

These results show that the composition of salt-tolerant microorganisms in different directions from the Salt Lake varies, which may be related to the specific physical and chemical characteristics of the soils around the Salt Lake. At the same time, with changes in distance from the lake shore the composition of salt-tolerant microorganisms also varied significantly. Changes in microflora represent variations the function and metabolic capabilities. Therefore, we analyzed the functional metabolism and gene regulation of different microbial groups to determine the salt tolerance mechanism of salt-tolerant bacteria.

### Weighted gene coexpression network analysis

**FIGURE 4:**
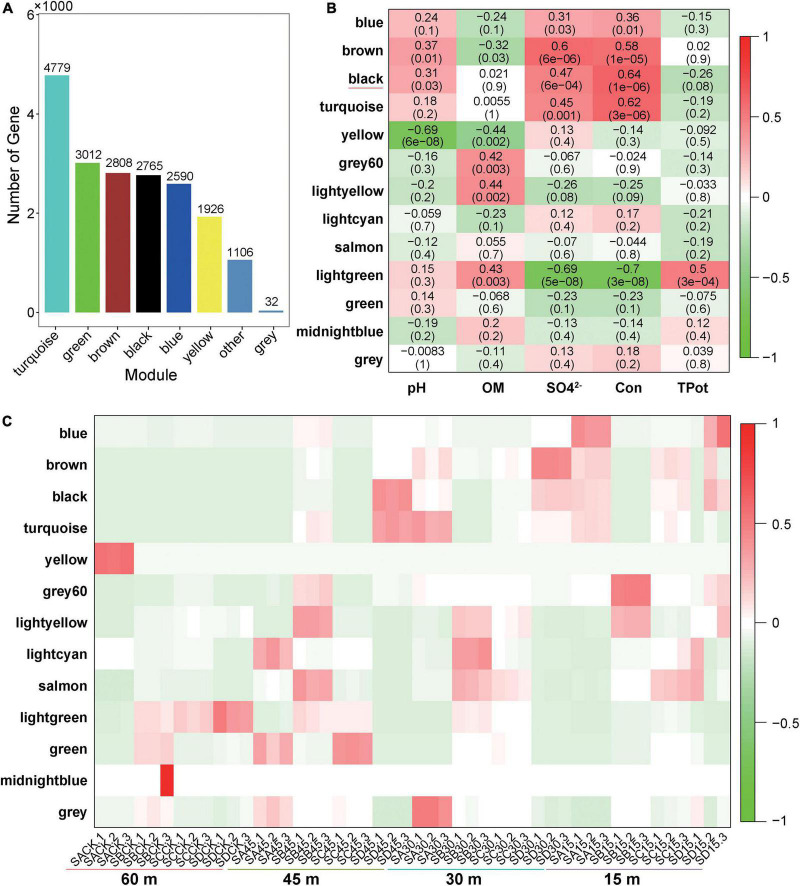
Correlation analysis of phenotypic traits and screening of key regulatory genes. **(A)** Represents the number of genes in each module divided based on WGCNA (weighted gene coexpression network analysis); **(B)** represents the correlation heatmap of the soil phenotype and each module, the leftmost color block represents the module, the rightmost color bar represents the correlation range, and the abscissa is the soil phenotype index. The darker the color in the figure is, the higher the correlation; red indicates a positive correlation, and green indicates a negative correlation. The number in each cell indicates correlation and significance, **(C)** expression pattern of all modules in the sample.

**FIGURE 5:**
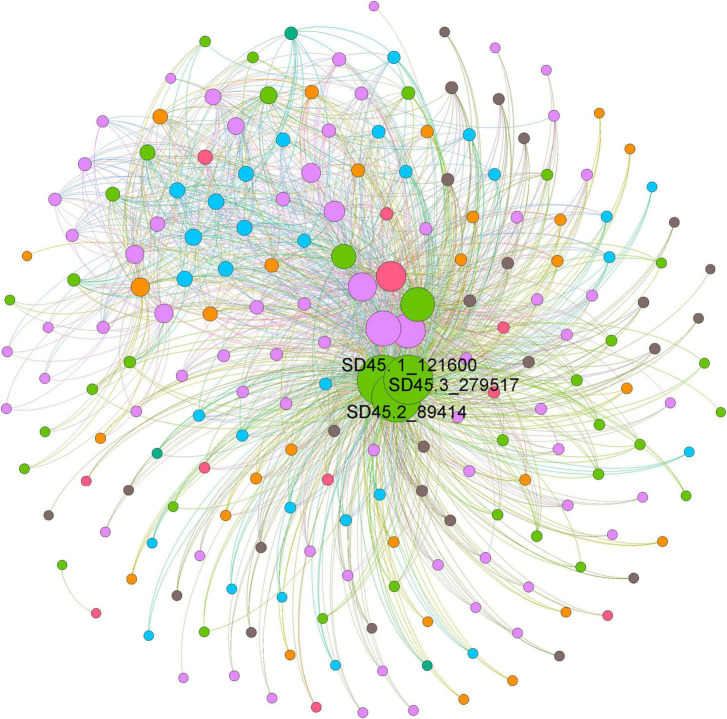
The coexpression of genes in the black module. Based on the similarity between genes in the key module, the module network was constructed, and the core genes were screened. Different colors represent genes from different samples, and the size of the circle represents the degree.

The functions of microorganisms are often closely related to genes. We used WGCNA to: identify gene modules with similar expression patterns; analyze relationships between gene sets and sample phenotypes; develop a map of the regulatory network between genes in grouped gene sets; and, identify key regulatory genes. First, to construct the co-expression network, the genes were divided into different modules, and a gene clustering tree was created. The genes close to each other (clustered on the same branch) were divided into the same module (Supplementary Figure 2), and the number of genes divided by each module (turquoise, green, brown, black, etc.) was 4,779, 3,012, 2,808, 2,765, etc. (Figure 4A). Next, we calculated the correlation and significance between each module and soil phenotype (Figure 4B) and found that the black module had the highest correlation (*r* = 0.64) with conductivity using the correlation heatmap. Therefore, we focused on the black module in the follow-up. Through the analysis of the expression patterns of all modules in the sample (Figure 4C), we found that several modules were like the black pattern, such as brown and turquoise. The closer to the Salt Lake shore, the higher the expression abundance. Therefore, we next showed the gene expression patterns of all samples in the black module in detail (Supplementary Figure 3). The abundance was low far from the lake shore and gradually increased with proximity to the lake, especially at 45, 30, and 15 m to the southeast. In the above analysis, we found that conductivity had a significant correlation with the black module and changed significantly with distance from the lake shore. Therefore, we focused on the analysis of KEGG enrichment in the black module (Table 1) and found that most were functionally expressed related to bacterial salt resistance, including functions related to transport and metabolism. In addition, based on the similarity between genes in the key modules, we constructed the key module network, explored the gene co-expression in the black module (Figure 5), screened the core genes based on the key modules, and found that SD45.2_89414, SD45.3_279517, and SD45.1_121600 were the hub genes.

**TABLE 1:** The KEGG enrichment of the gene in black module.

Pathway	Gene number	*P*- value	*Q*-value
Ribosome	105	0.00	0.00
Oxidative phosphorylation	96	0.00	0.00
ABC transporters	90	0.00	0.01
Pyruvate metabolism	62	0.04	0.39
Carbon fixation pathways in prokaryotes	58	0.04	0.39
Citrate cycle (TCA cycle)	47	0.10	0.61
Quorum sensing	58	0.13	0.63
Glycolysis/Gluconeogenesis	47	0.13	0.63
Microbial metabolism in diverse environments	220	0.19	0.73
Carbon metabolism	131	0.32	0.93
Metabolic pathways	590	1.00	1.00
Biosynthesis of secondary metabolites	262	1.00	1.00
Biosynthesis of amino acids	112	0.64	1.00
Glyoxylate and dicarboxylate metabolism	48	0.54	1.00
Purine metabolism	46	0.62	1.00

### Analysis of the salt tolerance mechanism

**FIGURE 6:**
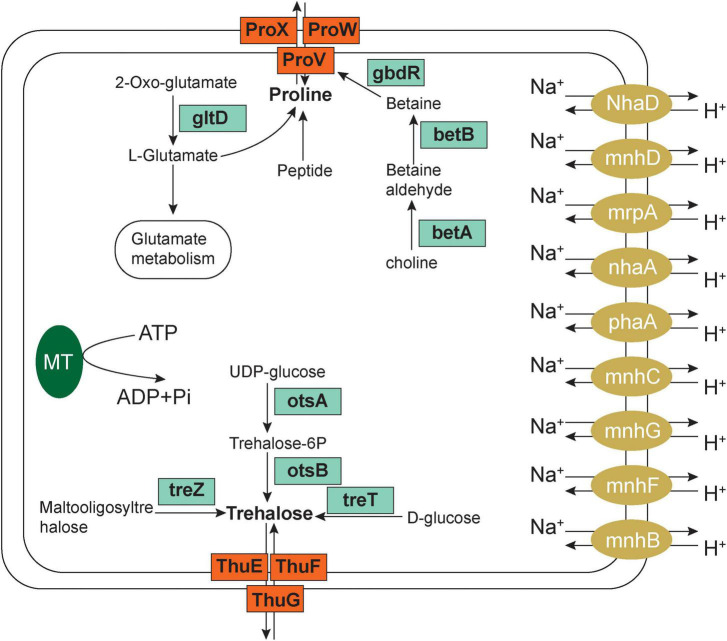
Salt tolerance-related pathways and mechanisms of salt lake microorganisms.

We then explored the salt tolerance using the KEGG annotation gene and found that the salt tolerance mechanism of microorganisms in the soil near the Salt Lake included increased regulation of Na^+^ efflux-related genes and organic compatible solute transport and biosynthesis (Figure 6).

The first element is the Na^+^ efflux mechanism: in this experiment, the expression of the Na^+^/H^+^ antiporter in saline alkali soil Groups at 15, 30, and 45 m increased, the multicomponent Na^+^/H^+^ antiporter subunit mnhA-G was upregulated, the cytoplasm participated in H^+^ absorption to exchange Na^+^, and cells extruded sodium to exchange external protons. Damage to cell membranes caused by salt stress can allow the transport of many extracellular salt ions into the cell. Excessive accumulation of Na^+^ causes toxicity to microbial cells. Current understanding suggests that the Na^+^/H^+^ antiporter is a transmembrane protein responsible for ion exchange. Driven by ATP, it can pump excess Na^+^ out of the cell to maintain the normal salt concentration and pH homeostasis of the cell.

The second element of salt tolerance involves organic-compatible solute transport and biosynthesis; the accumulation of osmotic protective substances is the most common mechanism for microorganisms to tolerate high salt environments. On the one hand, it can alleviate high salt pressure; on the other hand, these affinity solutes can be synthesized and degraded rapidly. This study found that the metabolic synthesis and related pathways of betaine, trehalose, proline, and glutamate increased, which collectively function in alleviating high salt stress.

#### Increased betaine synthesis

The proX gene encoding the glycine betaine/proline transport system substrate binding protein (K02002) and the proV and proW genes encoding the betaine/proline transport system ATP binding protein (K02000) were significantly upregulated in high salt environments according to the KEGG pathway annotation (Supplementary Figure 4A). In addition, this study found that the key genes betA and betB encoding choline dehydrogenase (K00108) and betaine aldehyde dehydrogenase (K00130) were significantly upregulated as well (Supplementary Figure 4B), and that betA and betB are currently the most effective genes related to salt tolerance. Choline dehydrogenase is an aldehyde protein that first converts choline into betaine aldehyde, and then betaine aldehyde dehydrogenase converts betaine aldehyde into betaine. The discovery of choline dehydrogenase (K00108) and betaine aldehyde dehydrogenase (K00130) indicate that the microorganisms in this study use the biosynthetic pathway of choline to betaine and, using upregulation of the two key pathways betA and betB, they achieve the synthesis of betaine, providing part of the overall salt tolerance mechanism.

#### Increased trehalose synthesis

Under environmental stress, such as high salt, drought, low temperature, or osmotic stress, plant cells and bacteria can use trehalose as an osmoprotective agent. There are five trehalose biosynthetic pathways in bacteria, including tre S, ots A/ots B, tre P, tre T, and tre Y/tre Z. In this study, we found three enzymes involved in the trehalose biosynthesis pathway. They are ots A/ots B (K00697, K01087), tre T (K13057), and tre Z (K01236). otsA is an enzyme that catalyzes synthesis of trehalose-6-phosphate, and otsB is a trehalose-6-phosphate phosphatase, which catalyzes the synthesis of trehalose-6-phosphate phosphorus. TreZ encodes malt oligos sugar trehalose hydrolase, and TreT encodes trehalose synthase. We also found that microorganisms in high-salt environments upregulate treT (K13057) and thuE (K10236), thuG and sugB (K10238), thuF and sugA (K10237) expression, which inhibits salt stress, thuF, sugA, while thuG and sugB encode trehalose/maltose transport system permeation proteins, and thuE encodes trehalose/maltose transport system substrate binding protein, indicating that with an increase in salinity, salt-tolerant microorganisms initiate the above four pathways, synthesizing trehalose to resist an external high-salt environment. In addition, the new related synthetic pathways (thuE, thuF, thug) and differential pathways found in this study provide new ideas and research directions for studying trehalose synthesis as a mechanism for managing salt tolerance.

#### Increased proline metabolism and synthesis

Proline is used as an osmotic pressure protection substance, and microorganisms can accumulate a large amount of proline as a stress protector when facing a high-salt environment. We found that with an increase in salinity the expression of genes encoding glycine betaine/proline transport system enzymes, such as proX (K02002), proV (K02000), and proW (K02001), was significantly upregulated. In addition, the expression of proline dehydrogenase PRODH (K00318), proline imine peptidase pip (K01259), and sodium/proline transporter putP (K11928) increased significantly. This suggests that when environmental salt stress increases, in addition to the synthesis of betaine and gene upregulation, it also promotes an increase in the expression of dehydrogenase and sodium transporter genes in an elegant combination of metabolic pathways that collectively resist the osmotic threat of a high-salt environment.

#### Glutamate metabolism and synthesis

Upregulation of the expression of gltD (K00266) glutamate synthase (NADPH/NADH) small chains, glutamate synthase (K00284), capA, pgsA (K07282) and cpg (K01295). CapA encodes γ-polyglutamic acid biosynthetic protein, and cpg encodes glutamate carboxypeptidase. This indicated that when salinity increases, microorganisms upregulate the glutamate synthase system to increase the concentration and content of glutamate in cells to resist the high salt environment.

In conclusion, when the external salt concentration is too high, microorganisms in Yuncheng Salt Lake increase the expression of the Na^+^/H^+^ antiporter by upregulating the Na^+^/H^+^ antiporter subunit mnhA-G to respond to salt stress, simultaneously upregulating the proV, proW, betA, and betB genes in the betaine/proline transport system to promote the conversion of choline into betaine, and upregulating ots A/ots B, tre T, tre Z and thuF, thuG and thuE, which encode the trehalose/maltose transport system to promote the synthesis of trehalose. The expression of PRODH, proline imine peptidase Pip and sodium/proline transporter putP encoding proline dehydrogenase increased, upregulated the gene expression of gltD, capA, pgsA and cpg, upregulated the biosynthesis of metabolites proline and glutamate and combined metabolic pathways to resist a high salt penetration environment.

## Discussion

In this study, mategenomic technology was used to identify the microorganisms in the soil at 15, 30, and 45 m in Yuncheng Salt Lake, Shanxi Province, allowing analysis of the species composition, functional expression, and salt tolerance mechanisms of microorganisms using KEGG pathways. The correlation between phenotypic traits and gene modules was further analyzed based on WGCNA to find and screen key regulatory genes. The results showed that the community structure of salt-tolerant microorganisms changed gradually with distance from the Yuncheng Salt Lake shore. Compared with soil not influenced by the salt content of the Salt Lake, the relative abundance of salt-tolerant *Gemmatimonas* and *Nitriliruptor* increased significantly. The two salt-tolerant bacteria found in this study are consistent with the conclusions of Yue et al. (2020). They revealed changes in the function and structure of microorganisms in the rhizosphere and soil of saline alkali land by using amplicons and macrogenome sequencing to investigate the genes of *Nitriliruptor* and *Gemmatinomonas*. We identified microorganisms that are highly sensitive to salt stress and may play a key role in saline soil, as well as the important functional role of the two salt-tolerant bacteria in the microbial communities in response to salt stress.

Based on the correlation analysis between phenotypic traits and gene modules of WGCNA, we found that electrical conductivity in soil had the highest correlation with the black module, and the gene expression in the black module gradually increased in abundance near the lake, revealing a salt tolerance mechanism. SD45.2_89414, SD45.3_279517, and SD45.1_121600 were identified as hub genes by further screening key regulatory genes of the black module.

Halophilic microorganisms evolved in high salt environments. To understand resistance to the high osmotic pressure present in high salt environments (Sun X. et al., 2021), we investigated mechanisms halophilic bacteria use to adapt to their extreme environment. At present, there are two mechanisms used by halophilic bacteria to cope with high salt: the first is the internal salt mechanism. There is an H^+^ pump in the purple membrane of the cell membrane of halophilic bacteria, which endows these bacteria with the function of Na^+^ reverse-transport, which forces salt ions to move from the inside of the cell to the outside (Plemenitaš et al., 2016). Halophilic bacterial cells maintain a high internal concentration of K^+^. These attributes allow halophilic bacteria to maintain the activity of enzymes and proteins, effectively prevent salt-induced aggregation between protein molecules and enzymes and playing a key role in the intracellular enzyme system (Meng et al., 2019). This mechanism exists in most halophilic archaea and anaerobic halophilic bacteria. In this study, we found that the expression of the Na^+^/H^+^ antiporter increased, which can pump excess Na^+^ out of cells and maintain normal salt concentrations and pH homeostasis. A second, salt-protecting tool is the compatible solute exocytosis mechanism, which means that halophilic bacteria remove salt from the cytoplasm and produce protective internal solutes to resist the high osmotic pressure outside the cell. Under hypertonic conditions, halophilic bacteria accumulate high concentrations of these protective substances inside their cells, while under hypotonic conditions, compatible substances can be quickly discharged from the cell or catabolized. A compatible solute mechanism allows metabolic flexibility for halophilic bacteria to adapt to a high salt environment, so it exists in most halophilic bacteria, especially in moderate halophilic bacteria. At present, studies have shown that the most widely used organic solutes for halophilic bacteria to maintain osmotic balance are tetrahydropyrimidine and betaine (Abdel-Aziz et al., 2013; Anburajan et al., 2019; Weinisch et al., 2019; Deole and Hoff, 2020). Tetrahydropyrimidine and betaine are derivatives of amino acids. These substances can maintain low water activity in the cytoplasm in a high salt environment to maintain the normal activity of intracellular enzymes. Under different salt concentrations, the substances accumulated by the same halophilic bacteria varied slightly (Lai and Lai, 2011; Lai et al., 2014). When cultured in a high salt environment (more than 3.5% salt concentration), the main compatible substance accumulated by Haiomonas israeiensis is tetrahydropyrimidine, while the main compatible substance accumulated by Haiomonas israeiensis in low salinity culture is trehalose (Regev et al., 1990; Mimura et al., 2008). In this study, the expression of related metabolic genes and pathways of tetrahydropyrimidine did not change significantly, but the expression of genes related to betaine, trehalose, proline, and glutamate increased, indicating different salt tolerances in different regions. Halophilic microorganisms possess different mechanisms for metabolically coping with external high salt stress. Sun Y. et al. (2021) analyzed *Candidatus nitrosoccus* sp. sol14 in saline alkali soil with high salt and high pH using macrogenome technology. They reported that this microorganism responded to external environmental stress mainly through direct Na^+^ extension/H^+^ import and synthesis of betaine, trehalose, glutamate, and proline in extreme habitats, and its mechanism was similar to the salt tolerance mechanism found in this study. In addition to both crucial adaptation mechanisms mentioned above, gram-negative bacteria play a role in the process of osmotic regulation by exposing the proteins OmpC and OmpF on their outer membrane surface (Mimura et al., 2008). As a prominent group of extreme environment microorganisms, halophilic bacteria are widely used in the food industry, medical and pharmaceutical industry, and enzyme preparation production operations based on their special physiological and biochemical characteristics. The enzymes produced by halophilic bacteria typically require a specific salt concentration to maintain their activity (Raddadi et al., 2015). The newly identified isomerases and hydrolases produced by them maintain high activity even under extreme salt conditions. Thus, they are the primary source of salt-tolerant enzymes in industry. Similarly, salt-tolerant cellulase, xylanase, superoxide dismutase, and nuclease have been reported; they resist high salt conditions by accumulating compatible substances in the cytoplasm. These substances can be used, for example, as stabilizers, antifreezes, and cosmetic moisturizers. Therefore, studying the mechanisms of microbial salt tolerance is of enormous potential significance in the development of biosynthesis pathways for addressing environmental stress.

## Conclusion

In conclusion, this study explored the microbial community composition and salt tolerance mechanisms of salt-tolerant microorganisms in the soils surrounding Yuncheng Salt Lake, identified salt-tolerant N*itriliruptor* and *Gemmatinomonas bacteria*, and investigated their metabolic responses to external high salt stress through the mechanisms of Na^+^/H^+^ antiporter regulation and organic compatible solute biosynthesis (Betaine, trehalose, proline, and glutamate). These findings provide further insights into critically important mechanisms used by salt-tolerant microorganisms in coping with ionically extreme environments.

## Data availability statement

The datasets presented in this study can be found in online repositories. The names of the repository/repositories and accession number(s) can be found in the article/Supplementary material.

## Author contributions

GS conceived and designed the experiments. FZ, YZ, and DZ wrote original draft together. ZZ, QL, PM, and GZ analyzed the data. YW, SW, and SG provided experimental resources and methodology. All authors have read and approved the final manuscript.

## Supplementary material

Supplementary Figure 1 The changes in electrical pH **(A)** and conductivity **(B)** in soil at different distances (15 m, 30 m, 45 m, and 60 m) from the lake shore.

Supplementary Figure 2 The gene clustering tree. The upper part is the hierarchical clustering tree of genes, and the lower part is the gene module.

Supplementary Figure 3 The expression pattern of genes in the black module in all samples.

Supplementary Figure 4 **(A,B)** The expression of proX and betA of betaine in each group.
