# Supplementary material for: Metagenomic analysis of the soil microbial composition and salt tolerance mechanism in Yuncheng Salt Lake, Shanxi Province
Source: Front Microbiol. 2022 Sep 26;13:1004556. doi: 10.3389/fmicb.2022.1004556 (PMC9549588; doi:10.3389/fmicb.2022.1004556)
Supplement: Supplementary file 1 [file Table_1.DOCX]

Supplementary Table 1 The overview of metagenomic sequencing

| Sequencing data |  | Gene prediction |  |
| --- | --- | --- | --- |
| Total Raw Data | 313,696.71 Mbp | Total ORFs | 18,481,910 |
| Average Raw Data | 6,535.35 Mbp | Average ORFs | 385,040 |
| Total Clean Data | 313,272.29 Mbp | Gene catalogue | 10,336,833 |
| Average Clean Data | 6,526.51 Mbp | Complete ORFs number | 2,192,023 |
| Effective percent | 99.86% | Complete ORFs precent | 21.21% |
| Metagenome assembly |  | Total length (Mbp) | 5,643.98 |
| Scaffolds (Average) | 252,975 | Average length (bp) | 546.01 |
| Total length (nt) | 10,94.009249 Mbp | GC percent | 66.22% |
| Average length (nt) | 901.11 bp | Gene function annotation |  |
| Longest length (nt) | 179,315 bp | Gene catalogue | 10,336,833 |
| N50 length (nt) | 861.27 bp | Annotated on KEGG | 6,756,892(65.37%) |
| N90 length (nt) | 546.33 bp | Annotated on KO | 3,744,883(36.23%) |
| Species annotation |  | Annotated on KO number | 8,623 |
| Gene catalogue | 10,336,833 | Annotated on EC | 2,409,174(23.31%)/2,905 |
| Annotated on NR | 7,943,365(76.85%) | Annotated on pathway | 2,432,771(23.53%)/415 |
| Annotated on Unclassified | 29.84% | Annotated on eggNOG | 6,381,745(61.74%) |
| Annotated on Kingdom level | 70.16% | Annotated on OG | 6,381,745(61.74%)/49,643 |
| Annotated on Phylum level | 61.15% | Annotated on CAZy | 364,553(3.53%) |
| Annotated on Class level | 55.87% |  |  |
| Annotated on Order level | 50.67% |  |  |
| Annotated on Family level | 47.57% |  |  |
| Annotated on Genus level | 43.38% |  |  |
| Annotated on Species level | 34.47% |  |  |
