# Supplementary material for: Metagenomic analysis of the soil microbial composition and salt tolerance mechanism in Yuncheng Salt Lake, Shanxi Province
Source: Front Microbiol. 2022 Sep 26;13:1004556. doi: 10.3389/fmicb.2022.1004556 (PMC9549588; doi:10.3389/fmicb.2022.1004556)
Supplement: Supplementary file 2 [file Data_Sheet_1.docx]

**Supplementary materials**

Fig. S1 The changes in electrical pH(A) and conductivity(B) in soil at different distances (15 m, 30 m, 45 m, 60 m) from the lake shore.

Fig. S2 The gene clustering tree. The upper part is the hierarchical clustering tree of genes, and the lower part is the gene module.

Fig. S3 The expression pattern of genes in the black module in all samples.

Fig. S4 A-B The expression of proX and betA of betaine in each group.
