# Supplementary figures and images for: Metagenomic analysis of the soil microbial composition and salt tolerance mechanism in Yuncheng Salt Lake, Shanxi Province
Source: Front Microbiol. 2022 Sep 26;13:1004556. doi: 10.3389/fmicb.2022.1004556 (PMC9549588; doi:10.3389/fmicb.2022.1004556)

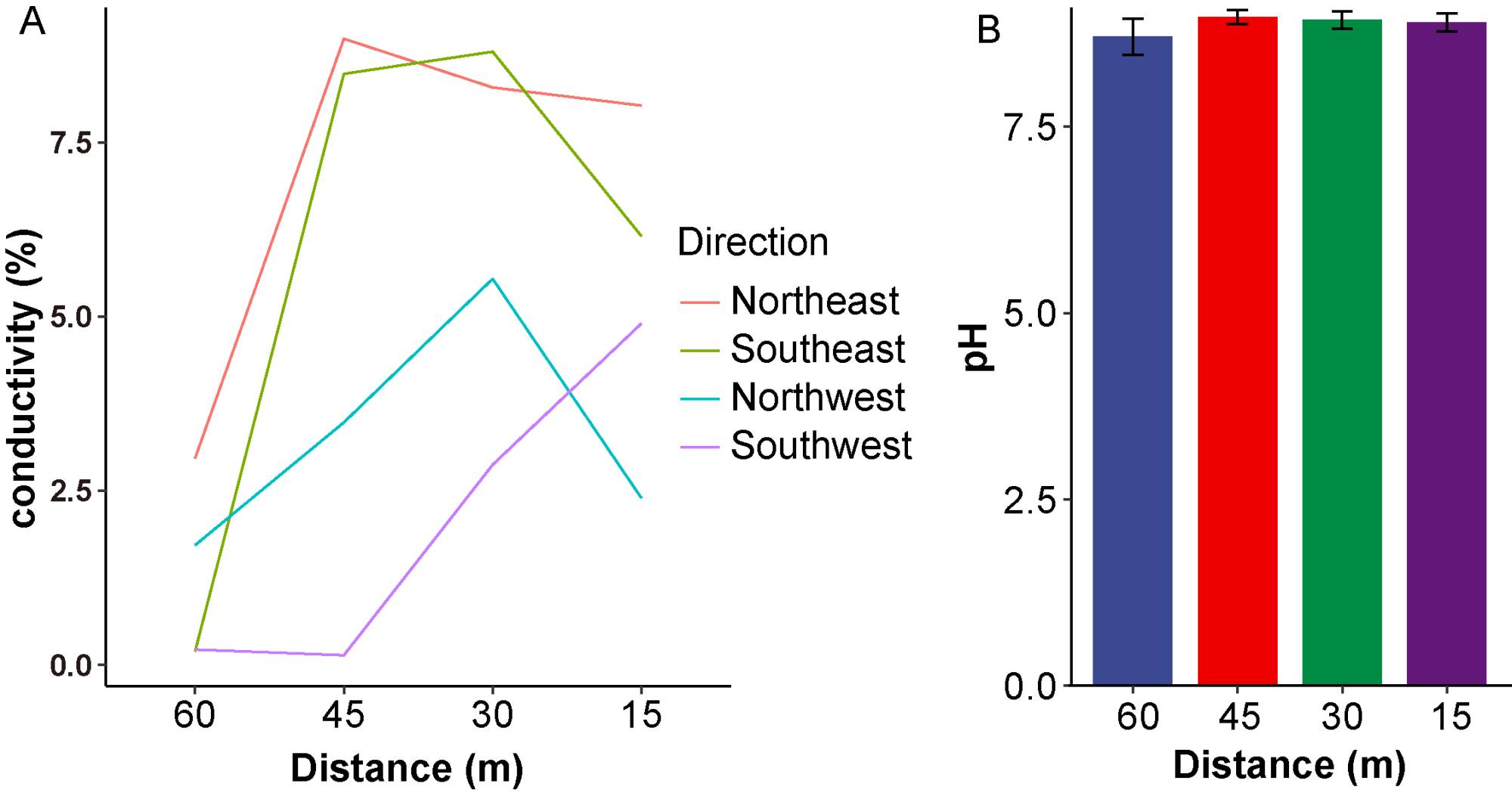

Supplement: Supplementary Figure 1 — The changes in electrical pH (A) and conductivity (B) in soil at different distances (15 m, 30 m, 45 m, and 60 m) from the lake shore. [file Image_1.JPEG]

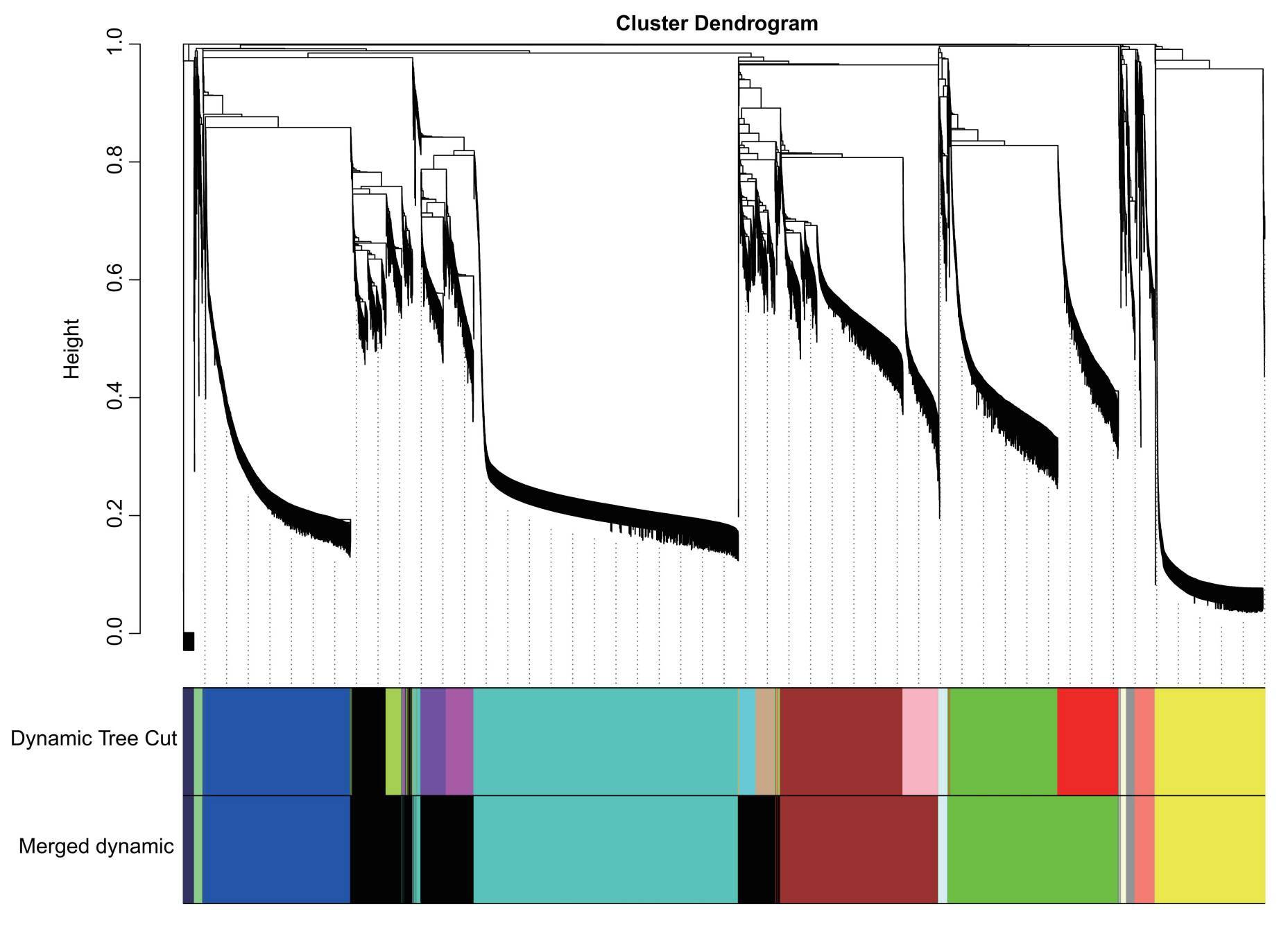

Supplement: Supplementary Figure 2 — The gene clustering tree. The upper part is the hierarchical clustering tree of genes, and the lower part is the gene module. [file Image_2.JPEG]

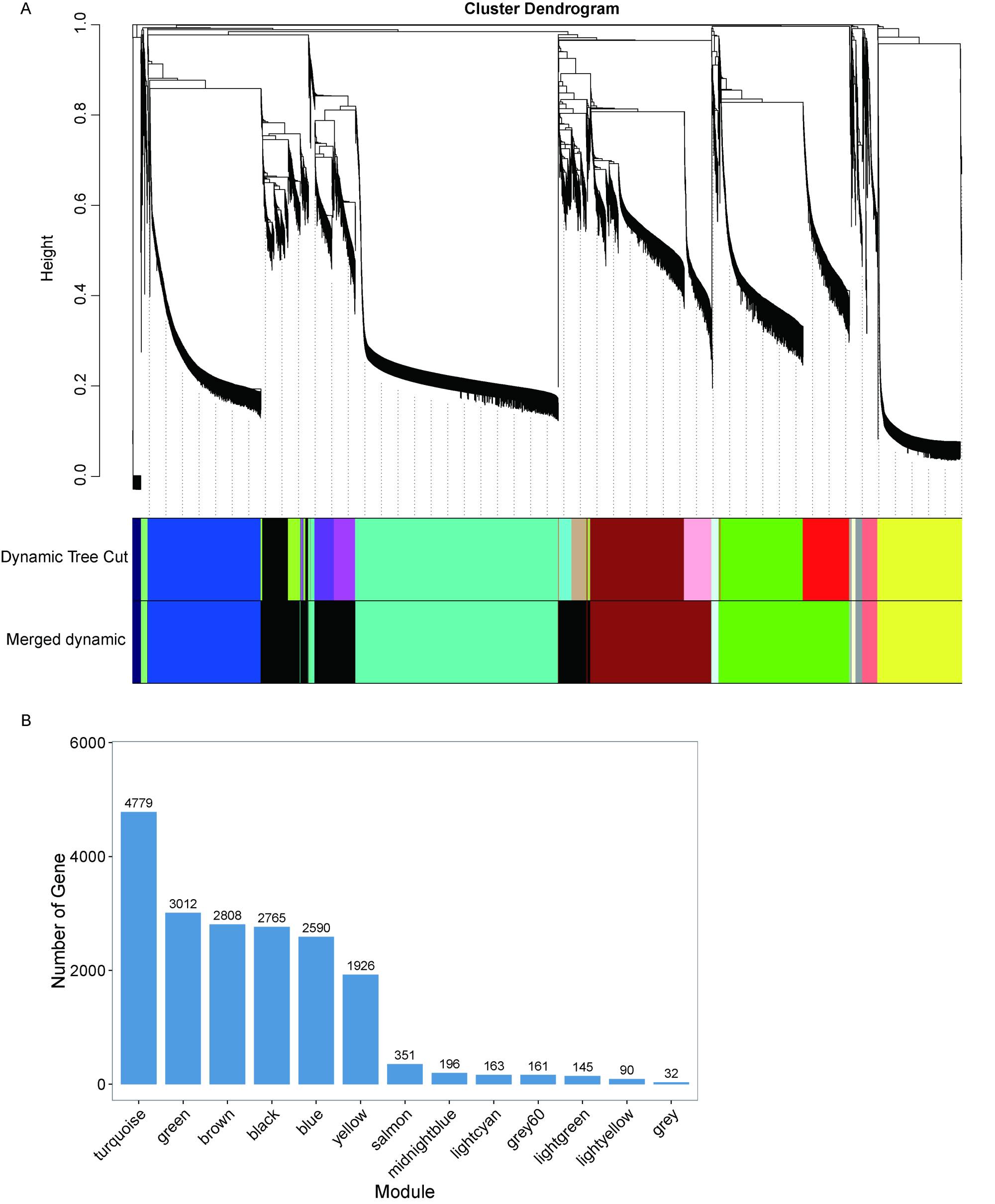

Supplement: Supplementary Figure 3 — The expression pattern of genes in the black module in all samples. [file Image_3.JPEG]

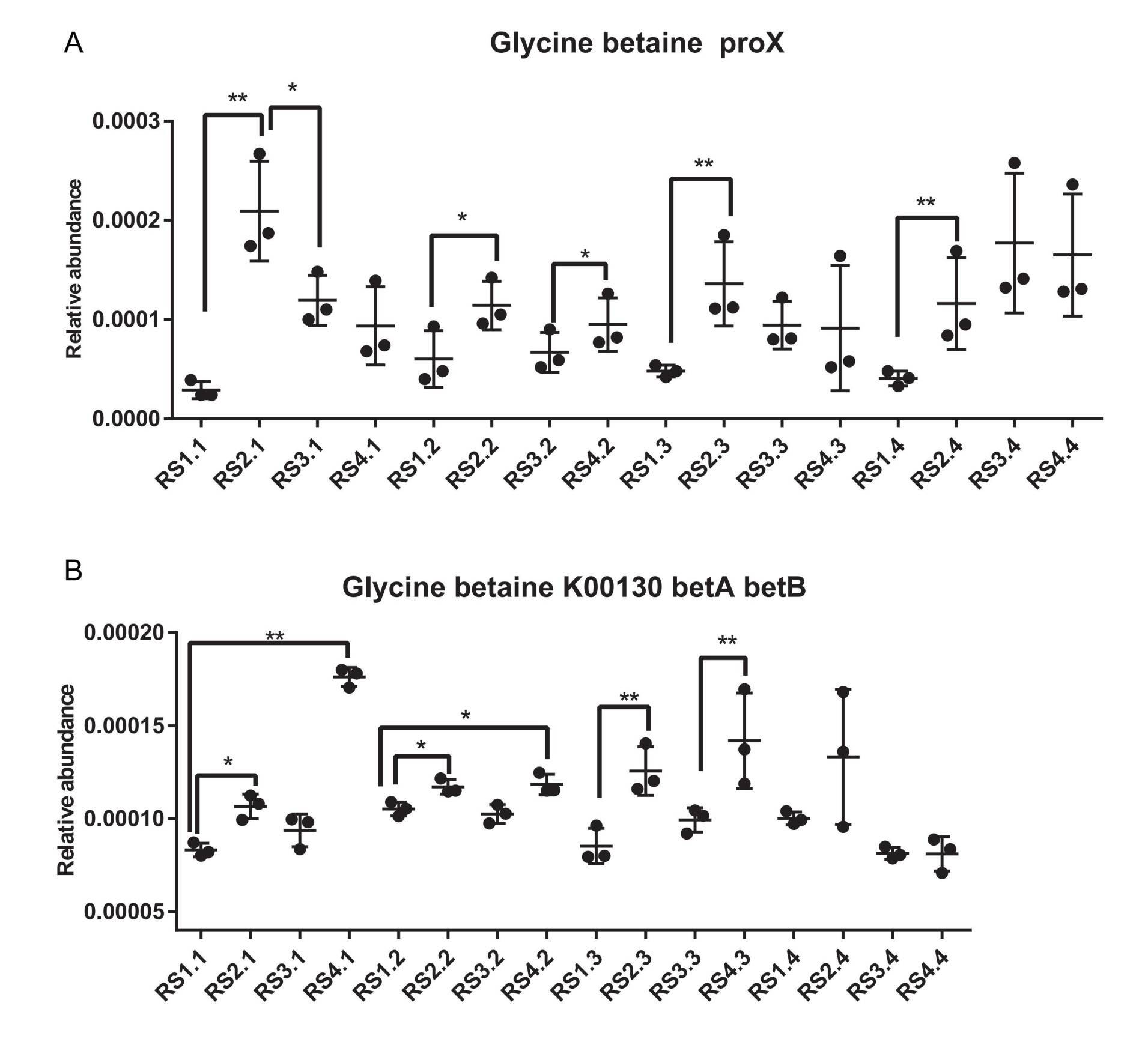

Supplement: Supplementary Figure 4 — (A,B) The expression of proX and betA of betaine in each group. [file Image_4.JPEG]
